# An Integration of Linkage Mapping and GWAS Reveals the Key Genes for Ear Shank Length in Maize

**DOI:** 10.3390/ijms232315073

**Published:** 2022-12-01

**Authors:** Zhenjuan Liang, Na Xi, Hao Liu, Peng Liu, Chenchaoyang Xiang, Chen Zhang, Chaoying Zou, Xuyujuan Cheng, Hong Yu, Minyan Zhang, Zhong Chen, Guangtang Pan, Guangsheng Yuan, Shibin Gao, Langlang Ma, Yaou Shen

**Affiliations:** State Key Laboratory of Crop Gene Exploration and Utilization in Southwest China, Maize Research Institute, Sichuan Agricultural University, Chengdu 611130, China

**Keywords:** maize, ear shank length, QTL mapping, association analysis, *Cyclin11*

## Abstract

Ear shank length (ESL) has significant effects on grain yield and kernel dehydration rate in maize. Herein, linkage mapping and genome-wide association study were combined to reveal the genetic architecture of maize ESL. Sixteen quantitative trait loci (QTL) were identified in the segregation population, among which five were repeatedly detected across multiple environments. Meanwhile, 23 single nucleotide polymorphisms were associated with the ESL in the association panel, of which four were located in the QTL identified by linkage mapping and were designated as the population-common loci. A total of 42 genes residing in the linkage disequilibrium regions of these common variants and 12 of them were responsive to ear shank elongation. Of the 12 genes, five encode leucine-rich repeat receptor-like protein kinases, proline-rich proteins, and cyclin11, respectively, which were previously shown to regulate cell division, expansion, and elongation. Gene-based association analyses revealed that the variant located in *Cyclin11* promoter affected the ESL among different lines. *Cyclin11* showed the highest expression in the ear shank 15 days after silking among diverse tissues of maize, suggesting its role in modulating ESL. Our study contributes to the understanding of the genetic mechanism underlying maize ESL and genetic modification of maize dehydration rate and kernel yield.

## 1. Introduction

Maize (*Zea mays* L.) is an important crop, which serves as animal feed, human food, and industrial raw materials [1]. Maize kernel yield is significantly affected by the transport efficiency of assimilates from photosynthetic source leaves to reproductive sink organs [2,3]. Ear shanks support ears and are special channels to transport photosynthetic products in maize via vascular bundle systems. Previous studies showed that ear shank length (ESL) has another major effect on mediating kernel dehydration rate (KDR) via husks in addition to that on influencing kernel yield. While KDR is a key factor influencing maize kernel quality, mechanized harvesting, storage, transportation, and further processing. Consequently, dissecting the important components that affect ESL is significant to promote mechanized production of maize [4,5].

Ear shanks are shortened branch stems, which are formed by the developed nodes and internodes differentiated from the bases of female ear growth cones. Plant hormones including abscisic acid, auxin and ethylene play significant roles in the development of vascular bundle system of maize ear shanks [6,7,8,9]. A previous study showed that the ESL is a quantitative trait and its narrow heritability was approximately 72.2% in diverse F2 populations [10]. Using the inbred lines with distinct ESLs, Ji et al. constructed F2 populations and revealed that the ESL has high additive and additive-dominance effects [11,12]. Liu et al. used three recombinant inbred line (RIL) populations to detect the quantitative trait loci (QTL) for maize ESL. Finally, 10 QTL were mapped to chromosomes 1, 2, 4, and 9, with the total phenotypic variation explained (PVE) ranging from 13.67 to 30.81% among the three populations. Each of these QTL had the PVE varying from 6.15 to 16.7%, suggesting that maize ESL was controlled by both minor- and major-effect genes [12]. A recent study dissected the genetic basis of 15 vascular system-related traits in maize ear shank using genome-wide association studies (GWAS), revealing 69 unique QTL and 348 candidate genes for these traits [13]. Using the mutagenesis method, the *ZmVLN2* gene was validated to mediate the ear shank diameter [13]. 

In addition to *ZmVLN2*, only a few causal genes were indicated to affect the development of ear shanks. *OPR7* and *OPR8* encode two oxo-phytodienoate reductases, and the double mutant *opr7 opr8* displayed an extreme ESL owing to the dramatically reduced jasmonic acid level [14]. Recently, a comparative transcriptome analysis was conducted to reveal the regulatory networks responsible for ear shank elongation in maize [11]. A total of 246 genes were identified as the commonly differentially expressed genes (DEGs) in different developing processes. Plant hormone signal transduction was among the top 20 significantly enriched pathways, which involved the genes participating in abscisic acid, brassinosteroids, cytokinin, ethylene, jasmonic acid, and salicylic acid signaling. These limited knowledge about genetic components and molecular mechanisms underlying ESL hindered the efficient genetic modification of KDR in maize.

Linkage mapping and GWAS are effective approaches for detecting the genetic architectures of quantitative traits in plants [15]. Traditionally, linkage mapping was employed to dissect the genomic regions controlling complex traits based on biparental populations [16]. Nevertheless, in linkage mapping, the QTL with minor effects are difficult to be identified because of its relatively low resolution in classical biparental populations [17]. Moreover, the lack of genetic diversity in segregation populations generally leads to the failure of rare allele identification [17]. On the contrary, GWAS provides a high-resolution method for QTL identification of quantitative traits, which is based on abundant genetic variations among diverse plant germplasms [18,19,20,21]. However, GWAS tends to cause higher false positive rates owing to the effects of population structures [22]. Therefore, the methods that combine linkage mapping and GWAS can complement the shortcomings of both methods and have been widely used to identify the genetic basis of agronomic traits in maize [15,23]. Furthermore, these genetic loci simultaneously detected using both methods provide more credible candidate genes for further studies of gene function. Cui et al. integrated linkage mapping and GWAS and revealed five candidate genes for husk morphology in maize [24]. Similarly, a combined association analysis with QTL mapping identified a locus that was associated with kernel shape in the segregation population and association panel [25]. Our previous study jointed GWAS and linkage mapping, eventually identifying seven microRNAs influencing maize kernel size. Among these microRNAs, miR164e was shown to regulate kernel development and control kernel size using miR164e-overexpression in *Arabidopsis* [15]. Up to now, there has been little research considering the genetic dissection of maize ESL using a combination of GWAS and QTL mapping.

In the present study, we performed a linkage analysis combined with association analyses on maize ESL in a biparental population and an association panel across three locations. The main objectives were to: (1) evaluate the phenotypic variation of the ESL in the segregation population and association panel, (2) identify the significant genomic regions responsible for the ESL using QTL mapping and GWAS, (3) detect co-localized genetic loci across different populations, and (4) reveal the key genes controlling the ESL by referring to the published transcriptome data and using gene-based association analysis. The results will provide a foundation for understanding the genetic mechanism underlying the ESL and contribute to molecular marker-assisted selection (MAS) of breeding maize varieties with high yield and KDR. 

## 2. Results

### 2.1. Evaluation of ESL Phenotype

**Figure 1:**
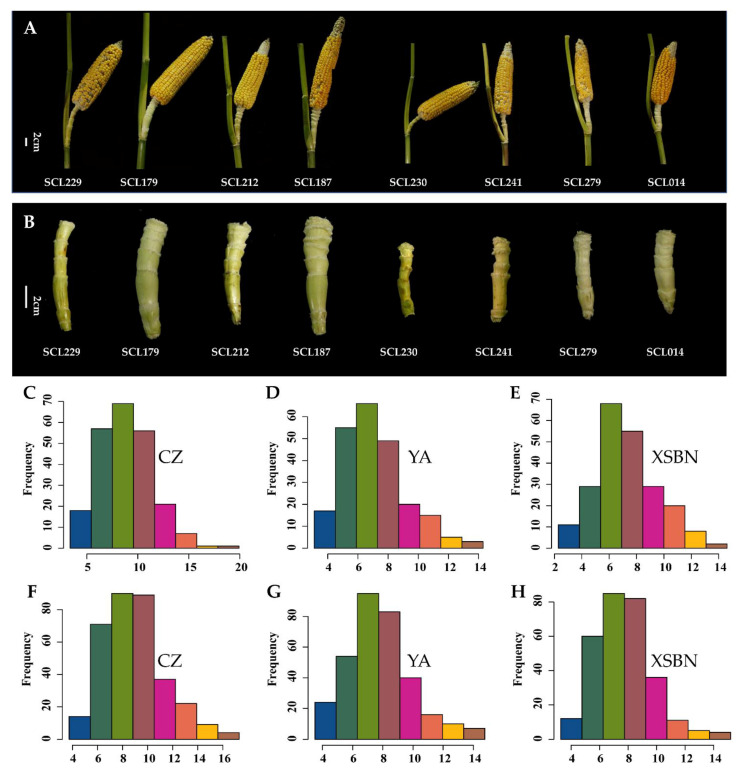
Frequency distributions of ear shank length. (**A**) Ears and shanks of different lines from the association panel, Scale bar, 2 cm; (**B**) Ear shank lengths of different lines from the association panel, Scale bar, 2 cm. (**C**–**E**) Frequency distribution of ear stank lengths in the IBM Syn 10 DH population. X-axis shows ear shank length (cm). (**F**–**H**) Frequency distribution of ear shank lengths in the IBM Syn 10 DH population. X-axis shows ear stank length (cm). CZ, YA, and XSBN represent the environments Chongzhou, Ya’an, and Xishuangbanna, respectively.

To systematically evaluate the ESL phenotype, we calculated the mean, standard deviation (SD), maximum, minimum, and coefficient of variation (CV) for the ESL in the segregation population and association panel across three environments, Chongzhou (CZ), Yan’an (YA), and Xishuangbana (XSBN). A *t*-test showed that the ESL was significantly (*p* < 0.01) different between the parents (B73 and Mo17) of IBM Syn 10 DH population under these environments except in XSBN (Table 1). In the IBM Syn 10 DH population, the ESL ranged from 3.37 to 19.93 cm, 3.10 to 14.30 cm, and 2.28 to 14.61 cm, with the mean value of 8.88 cm, 7.12 cm, and 7.28 cm in CZ, YA, and XSBN, respectively (Table 1). The SD was 2.55, 2.09, and 2.26, with the CVs of 28.73%, 29.33%, and 31.05%, in these locations, respectively (Table 1). In the association pool, the ESL ranged from 3.74 to 17.30 cm, 3.50 to 14.77 cm, and 3.27 to 15.19 cm, with the mean value of 8.93 cm, 7.77 cm, and 7.69 cm in CZ, YA, and XSBN, respectively (Table 1, Figure 1A,B). The SD was 2.45, 2.16, and 2.02, with the CVs of 27.47%, 27.74%, and 26.37%, in these environments, respectively. Taken together, these results showed that the two populations had abundant phenotypic variations regarding the ESL and was suitable to analyze the genetic basis of ESL. The ESL displayed continuous distributions among the two populations (Figure 1C–H), conforming to the characteristics of quantitative traits. Analysis of variance (ANOVA) displayed that the ESL was significantly (*p* < 0.01) different among the genotypes (G), environments (E), and genotype × environment interactions (G × E), for both populations (Table 1). Additionally, the broad-sense heritability ($H_{B}^{2}$) of the ESL was 0.81 and 0.82 in the IBM Syn 10 DH and association populations, respectively, suggesting that genetic factors play dominant roles in controlling maize ESL variations. 

### 2.2. QTL Responsible for ESL

**Figure 2:**
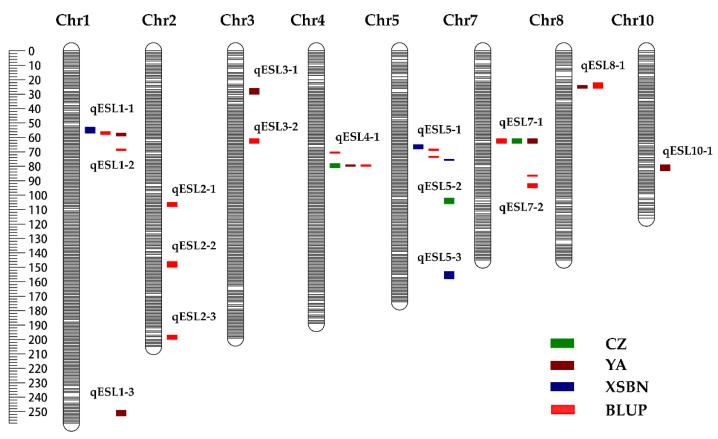
Chromosomal distributions of the QTL for ear shank length in the IBM Syn10 DH population in three environments. Chr1–Chr10 represent chromosomes 1–10. ESL denotes ear shank length. CZ, YA, and XSBN represent the environments Chongzhou, Ya’an, and Xishuangbanna, respectively. BLUP, best linear unbiased prediction.

To identify the QTL controlling maize ESL, we conducted a linkage mapping using the ESL phenotypic values of the IBM Syn 10 DH population across three environments and 6618 bin markers of this population. Finally, a total of 28 QTL with the PVE ranging from 3.39 to 9.80% were identified on all maize chromosomes except chromosomes 6 and 9 across three environments (Figure 2 and Table S1). These QTL had the logarithm of the odds (LOD) values varying between 2.51 and 6.69, among which 12 and 16 QTL showed the positively and negative additive effects, respectively (Table S1). No major QTL with the PVE > 10% was found, indicating that the ESL was mainly controlled by multiple minor-effect loci (Table S1). We regarded these QTL with physical interval smaller than 10 cm as the same QTL, and thus clustered the 28 QTL into 16 unique QTL (Figure 2 and Table S1). Of the 16 QTL, five were repeatedly detected across different environments or across environment(s) and BLUP. Specifically, three QTL, namely qESL1-1 (YA, XSBN, and BLUP), qESL4-1 (CZ, YA, and BLUP), and qESL7-1 (CZ, YA, and BLUP) were co-identified in two environments and using the BLUP values; two QTLs, namely qESL5-1 (XSBN and BLUP) and qESL8-1 (YA and BLUP) were repeatedly detected in a single environment and using the BLUP values (Figure 2 and Table S1). These repeatedly identified QTL were considered as the environment-stable QTL for ESL in this study. Notably, the qESL1-1 and qESL4-1 had the highest LOD values of 6.69 and 5.83, with the highest PVEs of 9.80% and 8.46%, respectively (Table S1).

### 2.3. Significant Associations for ESL

**Figure 3:**
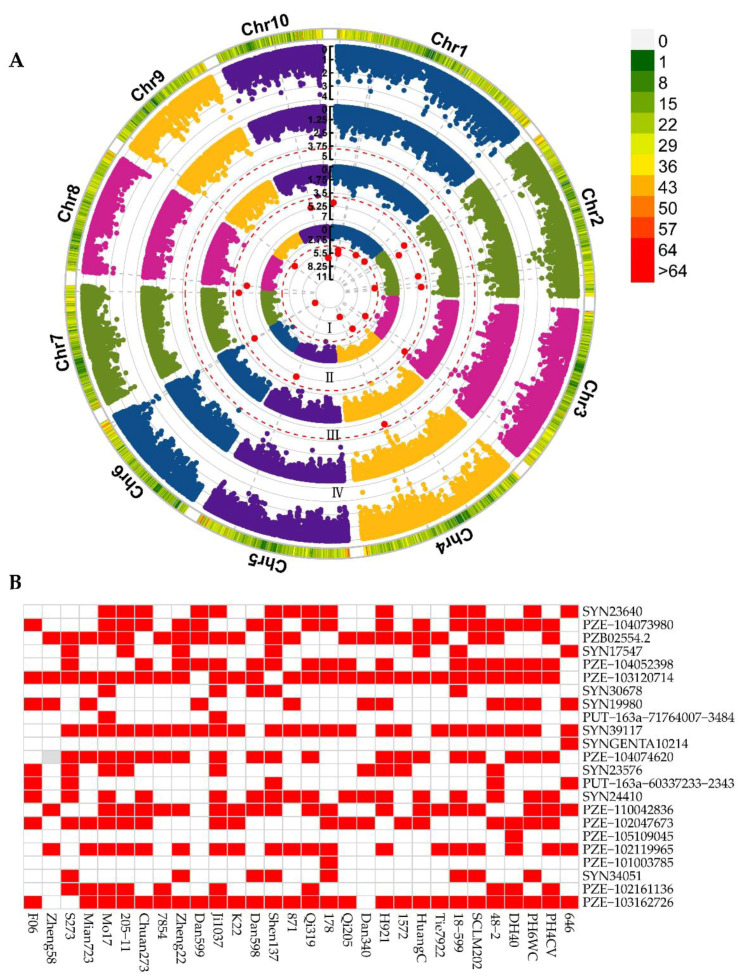
GWAS results and superior allele distributions. (**A**) Manhattan plots of genome-wide association study for ESL by using the FarmCPU model. (I) CZ, Chongzhou; (II) YA, Ya’an; (III) XSBN, Xishuangbanna; (IV) BLUP, BLUP, best linear unbiased prediction. Chr–Chr10 represent chromosomes 1–10; (**B**) Heatmap of superior allele distributions in the 30 elite lines. Red and white squares represent the superior and inferior alleles, respectively.

To reveal the significant genetic variations associated with ESL, we carried out a GWAS using the ESL phenotypes of this association panel across three environments and 56,110 SNP markers of this panel. After filtering SNP, a total of 43,817 high-quality SNPs were used for GWAS in this study. To identify the optimal model for GWAS, we compared the GWAS results among three models, namely the general liner model (GLM), mixed linear model (MLM), and fixed and random model circulating probability unification (FarmCPU) model. The results showed that the quantile-quantile (Q-Q) plot from the FarmCPU model was highly consistent with the expected distribution, which abruptly deviated from the expected *p*-value exclusively in the tail region (Figure S1). Therefore, the FarmCPU model was ultimately used to perform GWAS for the ESL. With a threshold of *p* = 1 × 10^−4^, 23 SNPs were significantly associated with the ESL across three locations (Table S2). These SNPs were distributed on all 10 chromosomes and explained 87.16% of the total phenotypic variations. No significant SNP was repeatedly detected in multiple environments, suggesting that environments exerted significant effects on the ESL. The SNP (SYN23640) that was situated on chromosome 6 had the lowest *p*-value (4.92 × 10^−11^), with the PVE of 5.59% (Figure 3A).

A total of 30 elite lines that have been serving as the parents of commercial cultivars were included in the association panel (Table S3), which allowed to predict the utilization of the superior alleles of these 23 SNPs during maize breeding. In the present study, the alleles associated with a higher and a lower ESL were designated as superior and inferior alleles, respectively. The ratio of the superior allele for each significant SNP that was calculated by the number of the lines containing the superior allele divided by the total line number ranged from 3.33% to 90% (Figure 3B and Table S3). Among these, 11 SNPs had the superior allele percentage > 50%, and the remaining 12 SNPs had that < 50%. Notably, the ratios of superior alleles for the three SNPs (SYN39117, PZE-103120714, and PZE-103162726) were ≥80%, whereas the six SNPs (PUT-163a-60337233-2343, SYN30678, PUT-163a-71764007-3484, PZE-101003785, PZE-105109045, and SYNGENTA10214) had superior allele ratios < 20%. To modify the ESL using MAS, these nine SNPs could be therefore given priority. Furthermore, each of the 30 lines contained the superior allele number varying between 5 and 15, of which 16 lines consisted of ≥11 superior alleles and the other 14 lines included < 11 superior alleles (Table S3). Especially, the lines Mo17 and Ji1037 had 15 superior alleles and Zheng58 had only 5 superior alleles (Figure 3B and Table S3), which was consistent with the large ESLs of Mo17 (9.91 cm) and Ji1037 (7.20 cm) and the small ESL (5.24 cm) of Zheng58. Therefore, integration of more superior alleles should be considered to increase the ESL of Zheng58.

### 2.4. Candidate Genes Co-Localized by QTL Mapping and GWAS

In general, the loci that are repeatedly detected in different populations with diverse genetic backgrounds are relatively stable and reliable [26]. We thus focused on the significant SNPs that were located in the QTL intervals, which were considered the loci simultaneously identified in both populations. Comparison of physical positions revealed that the four SNPs PZE-102119965, PZE-102161136, PZE-104052398, and PZB02554.2 were located in these QTL: qESL-2-1, qESL-2-2, qESL-4-1, qESL-10-1, respectively. Within the linkage disequilibrium (LD = 300 kb) region of the four SNPs, a total of 42 gene models were identified (Table S4). According to the Kyoto Encyclopedia of Genes and Genomes (KEGG) enrichment analysis, these genes were involved in flavone and flavonol biosynthesis, carbon pool by folate, photosynthesis, butanoate metabolism, porphyrin metabolism, plant hormone signal transduction, and etc (Table S5). The previous study reported the transcriptional dynamics during maize shank elongation at four developmental stages (L1: 1.01 ± 0.02 cm, L2: 1.98 ± 0.03 cm, L3: 3.02 ± 0.04 cm, and L4: 3.98 ± 0.03 cm) [11] (https://www.ncbi.nlm.nih.gov/sra/PRJNA738962, accessed on 1 August 2022). We then identified the DEGs with the absolute log_2_ fold change ≥ 2 between each comparison among the four stages. Finally, among the 42 genes, 12 were differentially expressed in at least one of the comparisons. According to the expression patterns (Table S6), these genes were clustered into four groups, namely groups I, II, III, and IV (Figure S2). Group I contained the *Zm00001d005298*, *Zm00001d005406, Zm00001d005404*, and *Zm00001d006679,* genes, which were continuously upregulated at L2 and L3 and then downregulated at L4. Group II involves *Zm00001d050403, Zm00001d026281*, and *Zm00001d005293*, they were upregulated at L2 and maintained stable expression levels at L3 and then were downregulated at L4. Group III included only one gene, *Zm00001d026297*, whose expression was decreased at L2, increased at L3, and then maintained unchanged at L4. *Zm00001d026282, Zm00001d006684*, *Zm00001d026306*, and *Zm00001d026306* belonged to group IV, which indicated an opposite expression pattern to group II. 

### 2.5. Intragenic Variations Affecting ESL

**Figure 4:**
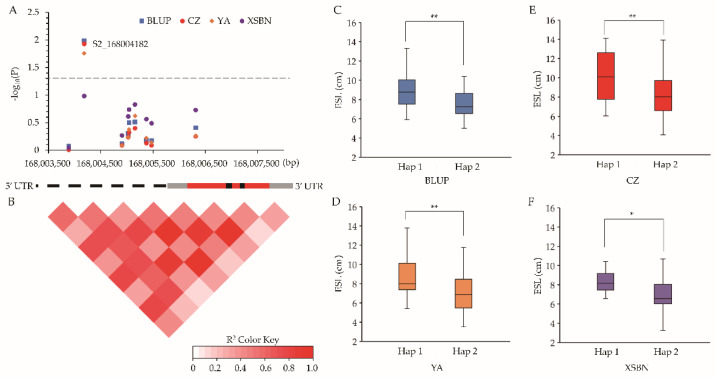
Results of *Zm00001d005293*-based association analysis (**A**) Association analysis of *Zm00001d005293*. The dashed line showed the significance threshold of *p* = 0.05 for detecting the associations. Schematic of the *Zm00001d005293* gene structure is shown in the bottom. (**B**) Pairwise linkage disequilibriums between markers. (**C**–**F**) Comparison of ESL between different haplotypes in BLUP (**C**), YA (**D**), CZ (**E**), and XSBN (**F**). CZ, YA, and XSBN represent the environments Chongzhou, Ya’an, and Xishuangbanna, respectively. BLUP denotes best linear unbiased prediction. Hap1, haplotype 1; Hap2, haplotype 2. * Significant at *p* = 0.05 level, ** Significant at *p* = 0.01 level.

According to the functional annotations of the 12 genes above, *Zm00001d005298* and *Zm00001d026306* encode leucine-rich repeat (LRR) receptor-like protein kinases [27]. *Zm00001d026282* and *Zm00001d026297* were annotated as proline-rich proteins (PRPs) [28], whereas *Zm00001d005293* encodes *Cyclin11* [29]. These proteins were previously reported to be involved in cell division, expansion, and elongation in plants. and were thereby considered the potential causal genes for the ESL. To further determine whether the variations within the five genes affected the ESL, gene-based association studies were conducted by using the genotypes of these genes and the ESL phenotypes of 77 maize lines randomly selected from the association panel. In terms of results, a total of 22 (21 SNPs and 1 InDel), 13 (12 SNPs and 1 InDel), 33 (32 SNPs and 1 InDel), 16 (11 SNPs and 5 InDels), and 59 (53 SNPs and 6 InDels) variants were detected in *Zm00001d005293*, *Zm00001d026282*, *Zm00001d005298*, *Zm00001d026297*, and *Zm00001d026306*, respectively (Table S7). Association analysis indicated that one SNP (S2_168004182, G↔T) in *Zm00001d005293* was significantly (*p* ≤ 0.05) associated with the ESL under the environments CZ and YA and using the BLUP values, this variant was located in the promoter region (Figure 4A,B). A *t*-test showed that the lines with the G allele had a significantly (*p* ≤ 0.05) larger ESL than those with the T allele (Figure 4C–F). However, no significant SNP was identified in the other four genes. Collectively, these results suggested that *Zm00001d005293* which encodes *Cyclin11* was the key gene affecting maize ESL.

### 2.6. Temporal and Spatial Expression Profile of Cyclin11

**Figure 5:**
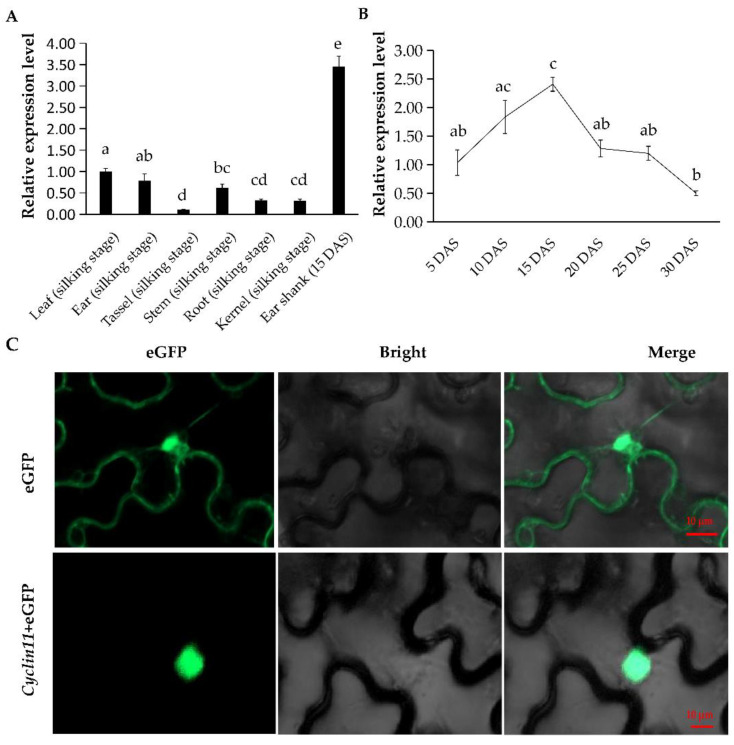
Temporal and spatial expression profile of *Cyclin11*. (**A**) *Cyclin11* expression level in different tissues of maize. Different small letters above the columns indicated significant (*p* ≤ 0.05) differences between various tissues. (**B**) *Cyclin11* expression level in different developmental stages of ear shanks. Different small letters above the columns indicated significant (*p* ≤ 0.05) differences between various developmental stages. (**C**) Subcellular localization of the *Cyclin11* protein in tobacco leaves, Scale bar 10 μm). DAS, day after silking.

To understand the potential role of *Cyclin11* in mediating the ESL, we dissected the expressions of *Cyclin11* in diverse maize tissues including roots, steams, leaves, ears, tassels, kernels, and ear shanks by using real-time quantitative PCR (RT-qPCR). Finally, *Cyclin11* displayed the lowest expression in maize tassels. However, ear shanks had the highest expression of *Cyclin11* among the seven tissues, followed by leaves and ears, implicating its crucial role in regulating ear shank development (Figure 5A). Moreover, we analyzed the expression levels of *Cyclin11* in ear shanks of a maize line (SCL212) with a large ESL at different developmental stages. As a result, the *Cyclin11* expression was gradually upregulated from 5 days after silking (DAS) to 15 DAS, and was then downregulated from 15 to 30 DAS, suggesting that *Cyclin11* played a significant role at the early stage of ear development (Figure 5B). To know the subcellular localization of the *Cyclin11* protein, we transformed the p35S:*Cyclin11*-eGFP fusion vector into tobacco leaves. The eGFP fluorescence signal was exclusively observed in the nucleus of the transformed leaves, indicating that the *Cyclin11* protein was localized in the nucleus (Figure 5C). 

## 3. Discussion

### 3.1. Combining the IBM Syn 10 DH Population and Association Panel to Detect the Genetic Basis of Maize ESL

High recombination frequencies and abundant phenotypic variations in linkage mapping populations are helpful to dissect the genetic basis of target traits [30]. The IBM Syn 10 DH population was derived from the cross of the parent lines B73 and Mo17, which shows a distinct difference in the ESL across different environments (Table 1). The CVs of ESL in this population were approximately 0.3 across three environments. Furthermore, this doubling-haploid (DH) population was constructed by six additional generations of open-pollination and haploid-doubling of the IBM Syn4 population. Compared with other RIL populations, the IBM Syn 10 DH population showed a higher frequency of genetic recombination and a shorter genetic distance between two adjacent markers [15]. These facilitate the fine-mapping of QTL for the ESL, for example, five QTL (qESL1-1, qESL1-2, qESL1-3, qESL5-3, and qESL7-1) were mapped to <1.0 Mb intervals and only 21–49 genes were contained in each of these regions. On the other hand, diverse genetic backgrounds and high heritability in association panels are beneficial to dissect the significant associations with target traits [31]. Based on the population structure, the association panel used in the present study was divided into the tropical, stiff stalk, and non-stiff stalk groups, which supported the high genetic diversity of this panel. The ESL ranged from 3.37 to 17.30 cm in this panel across three environments with the CVs being approximately 0.27. Moreover, the broad-sense heritability was 0.82 in this panel and was similar to that in the DH population, suggesting that the ESL was mainly controlled by genetic components. Using GWAS, a total of 23 SNPs were significantly associated with the ESL. Within the LD regions of these SNPs, several genes involved in cell division, expansion, and elongation were identified, including two proline-rich proteins, two LRR receptor-like protein kinases, cyclin11, and protein longifolia2 (Table S4). Finally, we identified 12 candidate genes localized in both populations, which were responsive to the development of maize ear shanks (Figure S2). All these findings verified the reliability of using the IBM Syn 10 DH population and association panel to detect the genetic basis of maize ESL.

### 3.2. Genetic Architecture of Maize ESL

To date, only a few studies focused on the genetic architecture of maize ESL. Liu et al. detected 10 ESL-related QTL in three maize RIL populations using linkage mapping [12]. In addition, Sun et al. performed a GWAS for maize ESL and uncovered 11 genetic regions controlling ESL in two environments [13]. In the present study, we detected 15 ESL-related QTL intervals using QTL mapping and identified 23 ESL-associated SNPs under three environments using GWAS. By comparing the results between these studies, we found that there were two overlaps of genetic regions between the previous and present studies. Specifically, the two ESL-related QTL (qSLCIK-1−1 and qSLBYD-2−1) identified by Liu et al. overlapped with the two QTL (qESL1-3 and qESL2-1) detected in our study, respectively [12]. Meanwhile, two ESL-associated SNPs identified in the present study were located in two ESL-related QTL identified by Liu et al., including PZE-101003785 (in qSLBYD-1−1) and PZE-102047673 (in qSLBYK-2−1) [12]. Furthermore, we revealed four ESL-associated SNPs residing in four ESL-related QTL intervals, respectively, in the present study (Table S4). Notably, in our study, five ESL-related QTL were repeatedly detected in multiple environments in linkage mapping. The above 13 genetic regions represented the population-common or environment-stable QTL and should be given priority in the identification of causal genes for maize ESL. However, the other QTL probably represented the population or environment-specific QTL, which was due to environmental effect and the difference in genetic backgrounds among various populations.

### 3.3. Candidate Genes Involved in Maize ESL

Cell division and expansion play decisive roles in cell number and size in a mature organ [32], and thereby control the size of an organ or a whole plant [33]. Combining QTL mapping, GWAS, and transcriptome analysis, we totally identified 12 population-common candidate genes for the ESL. Among them, *Zm00001d005298* and *Zm00001d026306* encode putative LRR receptor-like protein kinases, which have been demonstrated to play crucial roles in plant growth and development [34]. Overexpression of a LRR kinase in rice caused the dwarfism of the transgenic plants [27]. The receptor kinases located on the membrane can perceive the secreted peptides [35], considered as the essential mediators of communication between the cells controlling cell proliferation [36]. *Zm00001d026297* and *Zm00001d026282* were both annotated as PRPs, which were previously reported to play an important role in the process of plant growth and development [37]. EXTENSIN33, a proline-rich short elongation protein in Arabidopsis, affects hypocotyl elongation during the first stage of dark growth by regulating cell wall elongation [38]. In addition, the PRP-like1 participates in cell elongation and regulates root hair growth and development in Arabidopsis [37]. The candidate gene *Cyclin11* belongs to the D-type Cyclin (CYCD3) family, which contributes to cell division in plants [29]. CYCD is conserved in Arabidopsis, rice and poplar, which plays a central role in promoting cell division by responding to mitotic signals such as auxin and cytokinin [39]. In Arabidopsis, several CYCD subfamilies, including CYCD3; 1, CYCD3; 2, and CYCD3; 3, balance cell division and expansion inside branches by regulating mitosis and meristem responses to cytokinin [40]. Furthermore, CYCD regulates the development of vascular bundles by controlling the division of cambial cells [40]. Forzani et al. found that WOX5 controls cell division through CYCD3;3 in the cell quiescent center [41]. Remarkably, gene-based association analyses revealed that one variant in the promoter of *Cyclin11* was significantly associated with maize ESL across different environments. *Cyclin11* showed a higher expression in ear shanks than the other maize tissues. Moreover, *Cyclin11* was upregulated during ear development and its expression reached a peak on 15 DAS, which was consistent with the characteristics of ear shank elongation in maize. All these results supported that the five genes were potential casual genes for maize ESL, among which *Cyclin11* can be used to develop functional markers for genetic modification of maize ESL.

## 4. Materials and Methods

### 4.1. Plant Materials and Field Trials

In this study, an IBM Syn10 DH population that included 267 DH lines was used for linkage mapping of maize ESL [42]. An association panel consisting of 336 diverse inbred lines was employed to explore the associations with the ESL using GWAS [43]. The two populations were planted under three environments in China: Chongzhou (CZ, Sichuan Province, 30.32° N, 103.38° E, April 2021–August 2021), Ya’an (YA, Sichuan Province, 29.59° N, 102.57° E, April 2021–August 2021), and Xishuangbanna (XSBN, Yunnan Province, 22.02° N, 100.80° E, October 2021–March 2022). CZ has an average annual temperature of 16.0 °C and a total precipitation of 1132.0 mm in 2021; these values were 23.5 °C and 1024.4 mm in XSBN in 2021, and 17.5 °C and 1537.8 mm in YA in 2021. All the lines of the two populations were planted in completely random blocks with three replications. Each replication contained three rows, with the row length of 3 m and row spacing of 0.75 m. These materials were finally grown at a density of 62,000 plants/ha [44]. The field management was carried out according to the standard cultivation practices.

### 4.2. Sample Collection, Phenotyping and Statistical Analyses

**Figure 6:**
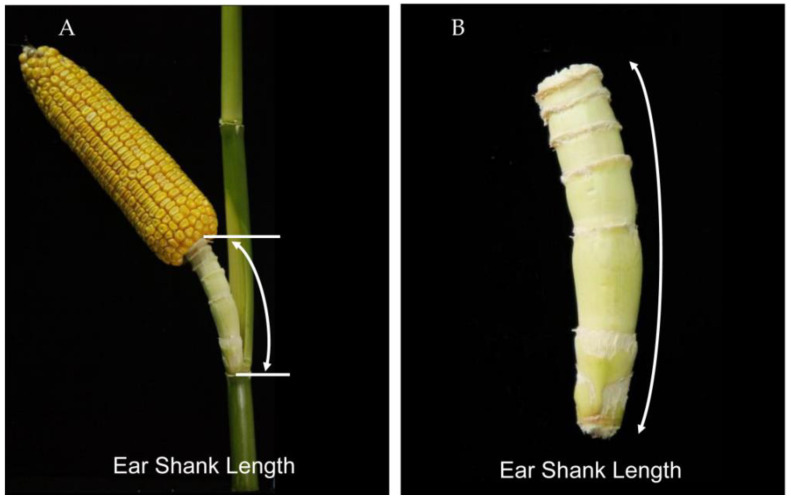
Schematic diagram of measurement of ear shank length. (**A**) Maize ear and its shank. (**B**) ear shank length examined in the present study. The white curves with arrows indicate the ear shank length.

For each plant, the ear with its shank was collected after ear maturity. Three ears with consistent growth were selected to measure the ESL for each line in a replication. The ESL was measured using a soft ruler, which was equal to the distance between the stem attachment point to the ear bottom [12] (Figure 6). The mean ESL of the three ears was designated as the phenotypic value for each replication and the average ESL across three replicates was used as the final phenotype per line in a single environment. 

IBM SPSS Statistics 27 software (https://www.ibm.com/analytics/spss-statistics-software, accessed on 21 July 2022) was used to analyze the phenotypic data, including mean, range, standard deviation (SD), coefficient of variation (CV), and variance. The broad-sense heritability ($H_{B}^{2}$) of the ESL was estimated as follows: $H_{B}^{2}$ = $\sigma_{g}^{2}/\left(\sigma_{g}^{2}+\sigma_{ge}^{2}/n+\sigma^{2}/nb\right)$ [45]. Herein, $\sigma_{g}^{2}$ is the genetic variation, $\sigma_{ge}^{2}$ represents the interaction between genotype and environment, n denotes the number of the environments, b shows the number of replications in each environment. The BLUP values of ESL across three environments were calculated using the lme4 package in the R (version 4.1.2) software, with the following formula:

BLUP = lmer (TRA ~ (1|LINE) + (1|LOC) + (1|REP %in% LOC) + (1|LINE: LOC)

### 4.3. QTL Mapping

A high-density genetic map with 6618 bin markers was previously constructed for the IBM Syn 10 DH population. The average distance between adjacent markers was 0.48 cM [46]. The WinQTLCart software [47]. (version:2.5, Wang S., North Carolina State University, Raleigh, North Carolina, accessed on 20 July 2022) was employed to perform linkage analysis based on the composite interval mapping (CIM). The walking speed was set to 1.0 cM and the LOD threshold was determined by the permutation test with 1000 repetitions [48]. When the peak distance between two QTL is less than 10 cM, they were defined as the same QTL [49]. The naming rule of QTL was as follows: q + ESL + serial number of chromosome + serial number of detected QTL. For example, in qESL-10-1, ‘q’ represents QTL, ‘ESL’ is the abbreviation of ear shank length, ‘10’ means chromosome 10, and ‘1’stands for the first QTL identified in chromosome 10.

### 4.4. Genome-Wide Association Study

The association panel was previously genotyped using the Illumina Maize SNP50 BeadChip, which contained 56,110 SNPs [43]. Genotype data were filtered with reference to the previously reported filtering criteria [48]. Finally, a total of 43,817 SNPs on 10 chromosomes were retained for GWAS after removing the SNPs with missing rate ≥ 20%, minor allele frequency (MAF) ≤ 0.05, or heterozygosity rate ≥ 20%. Herein, we tested three models to eliminate false positives and false negatives: GLM, performed in the Tassel software (version: 5.2.84, Peter J. Bradbury, New York, NY, USA); MLM, conducted using the GAPIT package in R (version 4.1.2); and FarmCPU, implemented using the FarmCPU package in R (version 4.1.2). In terms of results, among the three models, FarmCPU had the highest fit between the observed and predicted values of the Q-Q plot. Thus, FarmCPU was selected as the optimal model for GWAS of the ESL. The threshold of *p*-value for association identification was set to *p* = 1 × 10^−4^ [50]. All the gene models within the LD = 300kb regions of the significant SNPs were identified as the initial candidate genes from GWAS. The candidate genes were functionally annotated by referring to B73 RefGen V4 in MaizeGDB (https://www.maizegdb.org/, accessed on 25 July 2022).

### 4.5. DEG Identification

These genes that were simultaneously located within the LD regions of GWAS and QTL intervals of linkage mapping were extracted for the analysis of differential expression based on the published data of transcriptomes regarding maize ear elongation [11]. The significance threshold of DEG identification was set to the absolute log_2_ fold change = 2. The DEG expression patterns are clustered by the OmicShare tool, an online platform for data analysis and visualization (GENE DENOVO, Guangzhou, China, https://www.omicshare.com/tools/, accessed on 8 August 2022).

### 4.6. Gene-Based Association Analysis

According to the functional annotations, the DEGs involved in cell division, expansion, and elongation were subjected to gene-based association analyses using 77 lines randomly selected from the association panel. Genomic DNA was extracted from the leaves of the 77 lines using the CTAB method [51,52]. The full-length gene body and its 2000bp upstream of each candidate gene was individually amplified using PCR from the genomic DNA of each line. The amplification primer pairs were designed using the primer designing tool (https://www.ncbi.nlm.nih.gov/tool/primer-blast, accessed on 27 July 2022). The sequence analysis software DNAMAN (version 9.0, LynnonBiosoft, San Ramon, CA, USA) was used to align and analyze the sequences of the amplified fragments. The results of DNAMAN alignment were output in the Phylip format and then edited and modified using the BioEdit software (version 7.0.0, Tom H. Tampa, FL, Scotts Valley, CA, USA). The nucleotide polymorphisms among the 77 lines were analyzed using the DnaSP software (version 5.10.1, Julio R. Barcelona, Catalonia, Spain). After removing the variants with MAF < 0.05 or heterozygosity rate > 20%, the remaining SNPs and insertion/deletions (InDels) of each gene were used to detect the associations with maize ESL by GLM in Tassel (version: 5.2.84, Peter J. Bradbury, New York, NY, USA). The significance threshold of associated locus identification was set to *p*-value = 0.05. The haplotypes of the 77 lines were divided according to the significantly associated loci for each gene using the Haploview software [49]. A *t*-test was used to detect the significance of difference in the ESL between different haplotypes.

### 4.7. RT-qPCR

Seven tissues were collected from the line B73 and were subjected to expression analysis of *Cyclin11*, including roots (silking stage), stems (silking stage), leaf (silking stage), tassels (silking stage), ears (silking stage), kernels (6 DAP), and ear shank (15 DAS). Meanwhile, a line (SCL212) with long ear shank was subjected to analysis of *Cyclin11* expression patterns in ear shanks during different developmental stages. The ear shanks were sampled on 5, 10, 15, 20, 25, and 30 DAS, respectively. The above 13 samples were individually used for total RNA extraction with TRIzol Reagent (Invitrogen, Carlsbad, CA, USA). The first-strand cDNA was synthesized from approximately 2 µg of the total RNA with a reverse transcription kit (Novoprotein, Nanjing, China) according to the manufacturer’s instruction. The *Cyclin11* expression levels were quantified by conducting RT-qPCR with the SYBR qPCR SuperMix Plus (Novoprotein, Nanjing, China), the primers of RT-qPCR were listed in Table S8. *ZmActin* served as a reference gene, and the 2^−ΔΔCT^ method was employed to calculate the relative expressions of *Cyclin11* [53]. Three biological repetitions and three technical replicates were carried out for each sample. The significance analysis of relative expression levels was conducted by using the AVO function in R software (version 4.1.2). Multiple tests were performed using the LSD (least significance difference) method through variance analysis of different comparison groups.

### 4.8. Subcellular Localization

To understand the subcellular localization of *Cyclin11*, we cloned the full-length coding sequence of *Cyclin11* without the stop codon into the reporter vector pCAMBIA2300-eGFP, generating the p35S: *Cyclin11*-eGFP fusion expression vector. The fusion vector was then introduced into the *Nicotiana benthamiana* leaves for transient expression using the Agrobacterium-mediated method [53]. The p35S: eGFP vector served as the negative control. The eGFP signals in the transformed tobacco leaves were detected 48 h after culture under a confocal microscope (Zeiss LSM 800, Oberkochen, Baden-Württemberg, Germany).

## 5. Conclusions

In this study, QTL mapping and GWAS were combined to dissect the genetic basis and key genes controlling maize ESL. A total of 16 QTL were identified in the segregation population. Meanwhile, 23 SNPs were associated with the ESL in GWAS, of which four were located in the QTL identified by linkage mapping and were thus considered the population-common loci. In the LD regions of these common variants, 42 genes were identified and 12 of them were responsive to ear shank elongation. Of the 12 genes, five were involved in cell division, expansion, and elongation, which were designated as the key genes for maize ESL. Remarkably, *Cyclin11* showed the highest expression in 15 DAS-ear shank among diverse tissues, and a variant located in the *Cyclin11* promoter affected ESL among different lines. Our study contributes to the understanding of genetic mechanisms underlying maize ESL and the genetic modification of maize dehydration rate. 

## Figures and Tables

**Table 1:** Phenotypic description and analysis of variance for ESL in two populations under three environments.

		Population					
		IBM Syn 10 DH Population			Association Panel		
Env.		CZ	YA	XSBN	CZ	YA	XSBN
Mean (cm)		8.88	7.12	7.28	8.93	7.77	7.69
SD		2.55	2.09	2.26	2.45	2.16	2.02
Max (cm)		19.93	14.3	14.61	17.3	14.77	15.19
Min (cm)		3.37	3.10	2.28	3.74	3.50	3.27
CV (%)		28.73	29.33	31.05	27.47	27.74	26.37
Mean ± SD (cm)	B73	6.63 ± 0.64	3.5 ± 0.62	10.18 ± 0.98	-	-	-
	Mo17	11.14 ± 1.03	9.16 ± 0.61	9.42 ± 1.37	-	-	-
*p* value		0.006 **	0.0007 ***	0.56	-	-	-
F value E *		231.074 **			161.205 **		
F value G *		12.348 **			10.946 **		
F value G × E *		2.368 **			2.011 **		
Heritability		0.81			0.82		

Env., environment; SD, standard deviation; Max, maximum; Min, minimum; CV, coefficient of variation; CZ, Chongzhou; YA, Ya’an; XSBN, Xishuangbanna. * *p* ≤ 0.05; ** *p* ≤ 0.01; *** *p* ≤ 0.001. B73 and Mo17 are the parent lines of the IBM Syn 10 DH population.

## Data Availability

All data generated or analyzed during this study are available within the article or upon request from the corresponding author.

## Supplementary Materials

The following supporting information can be downloaded at: https://www.mdpi.com/article/10.3390/ijms232315073/s1.
