# Supplementary material for: An Integration of Linkage Mapping and GWAS Reveals the Key Genes for Ear Shank Length in Maize
Source: Int J Mol Sci. 2022 Dec 1;23(23):15073. doi: 10.3390/ijms232315073 (PMC9740654; doi:10.3390/ijms232315073)
Supplement: Supplementary file 1 [file ijms-23-15073-s001.zip › Review_ESL.Figure S1-S2.pdf]

International Journal of
Molecular Sciences

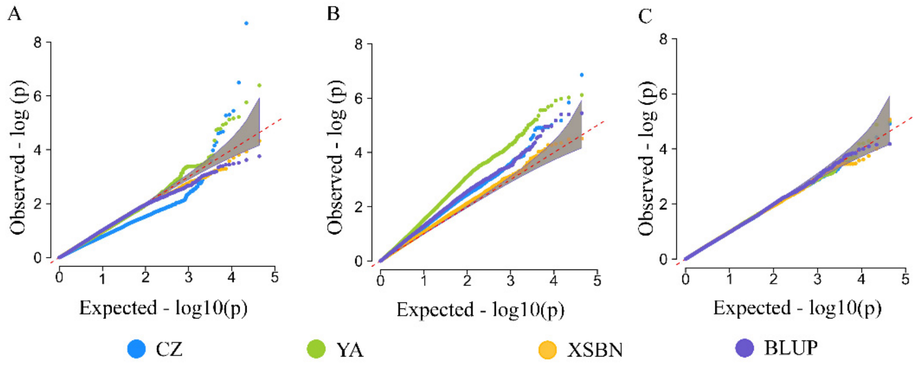

Figure S1. Q–Q plots resulting from three models for GWAS of ESL. (A) Q-Q plots of FarmCPU model; (B) Q-Q plots of GLM model; (C) Q-Q plots of MLM models.

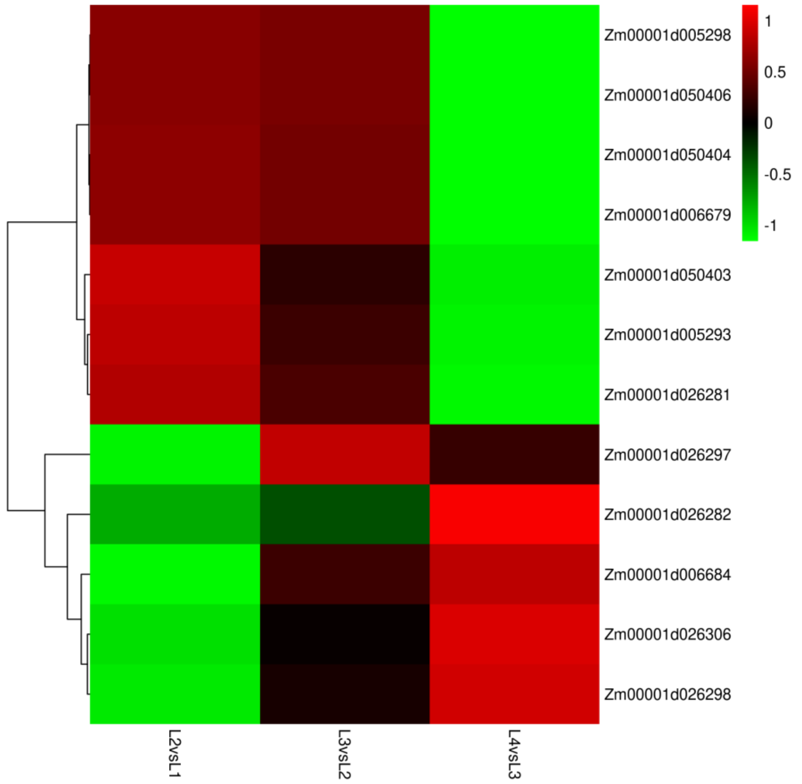

Figure S2. Heatmap of differentially expressed genes in four developmental periods
